# The burden of antimicrobial resistance at tertiary care hospital, southern Ethiopia: a three years’ retrospective study

**DOI:** 10.1186/s12879-019-4210-1

**Published:** 2019-07-05

**Authors:** Tsegaye Alemayehu, Mulubrahan Ali, Enkosilassie Mitiku, Mengistu Hailemariam

**Affiliations:** 1 School of Medical Laboratory Science, Hawassa University College of Medicine and Health Sciences, P.O.box 1560, Hawassa, Ethiopia; 2 Hawassa University Comprehensive Specialized Hospital, Hawassa, Ethiopia

**Keywords:** Antibiotics resistance, Clinical samples, Southern Ethiopia

## Abstract

**Background:**

Antibiotic resistance is a worldwide problem that crosses international boundaries and spread between continents easily. Hence, information on the existence of the causative microorganisms and their susceptibility to commonly used antibiotics are essential to enhance therapeutic outcome.

**Method:**

A cross-sectional study was conducted retrospectively at Hawassa University Comprehensive Specialized Hospital. The culture and antibiotic sensitivity data of the isolates were collected from the record books of the microbiology unit for the study period after official permission obtained from the institutional review board. The data entered and analyzed using statistical package for social science software version 20.

**Result:**

A total of 693 bacteria were retrieved, of these 435(62.77%) were gram-negative and the rest 258(37.23%) were gram-positive. Most of the isolates were from a urine sample. Among gram positives isolates, *S. aureus* and from gram negatives *Klebsiella spp* are the most recurrent isolate. Almost a remarkable resistance was observed to most of the antibiotics mainly, penicillin G (81.8%) and cotrimoxazole (81.1%), for gram-positive bacteria. The gram-negative bacteria also show resistance to ampicillin (92.5%), tetracycline (85%) and cotrimoxazole (93.1%).

**Conclusions:**

Nearly all isolate show substantial rates of resistance to most of the antibiotic that is frequently used in the study area. As already known we want to emphases on the importance of performing continuous monitoring of drug susceptibility to help the empirical treatment of bacterial agents to a health professional in the region. In addition, this data might help policymakers to control of antibiotics resistance.

## Background

Antibiotic resistance is a worldwide problem that can cross international boundaries and spread between continents with ease [1, 2]. The introduction of antibiotics in the mid-twentieth century was an important medical event in history with regard to reducing human and livestock morbidity and mortality. However, the subsequent and continuing intensive use of antibiotics since their introduction has helped to select a huge increase in the frequency of resistance among human pathogens [3]. Antibiotic resistance results in reduced efficacy of antibacterial agents make the treatment of patients difficult, costly, or even impossible. The impact on susceptible patients is most obvious, resulting in prolonged illness and even mortality. The magnitude of the problem and the impact of antimicrobial resistance (AMR) on human health including on costs for the health-care due to AMR still largely need further investigation [4].

The antibiotic resistance crisis has been attributed to the overuse and misuse of medications, as well as a lack of new drug development by the pharmaceutical industry due to reduced economic incentives and challenging regulatory requirements [5, 6]. In high-income countries, continued high rates of antibiotic use in hospitals contributed to selection pressure that has sustained resistant strains, forcing a shift to more expensive and more broad-spectrum antibiotics [6]. In low-income countries, divers antibiotic usage increases time to time due to high rates of hospitalization, and the high rate of infectious disease [6, 7].

On the other hand, the emergence of multidrug-resistant bacteria is a challenge for physicians to manage critical patients [8]. As already known, methicillin-resistant *s. aureus* (MRSA), extended-spectrum ß-lactamase (ESBL) *E. coli*, *v*ancomycin-resistant *s. aureus* and *enterococcus* associated morbidity and mortality are global problems [9, 10]. In regard to our setup, drug resistance to be expected as a major challenge even though there is no local report as well drug resistance monitoring system. Therefore, the aim of this study was to determine the burden of antibiotic resistance patterns by analyzing data collected retrospectively of a period of 3 years at the Hawassa University Comprehensive Specialized Hospital (HUCSH) in southern Ethiopia.

## Methods

This study design was an institution based cross-sectional study from November 2014–November 2017 retrospectively. The study was conducted on all clinical sample sent to the microbiology lab of HUCSH, Hawassa, Ethiopia. Hawassa is a city in Ethiopia, on the shores of Lake Hawassa in the Great Rift Valley. It is located 270 km south of Addis Ababa via Debre Zeit. The town serves as the capital of the Southern Nations, Nationalities, and Peoples’ Region (SNNPR). It lies on the Trans-African Highway to Cairo-Cape Town, and has a latitude and longitude of 7° 3′ 35 “ N and of 38° 28’ 11” E. It has an elevation of 1708 m above sea level [11].

The HUCSH serves as a referral centre for both public and private hospitals in South Regional State also for the neighbouring region. The hospital was established in November 2005 and serves about 12 million people in the Southern Nation, Nationalities’ and peoples’ region (SNNPR) and neighbouring Oromia. Besides providing clinical services, the hospital is a teaching hospital for medical and paramedical students. It is located 273 km south of the capital city, Addis Ababa. The microbiology laboratory at HUCSH was established in 2010. It is the only laboratory in the region that participates in obtaining accreditation.

Patient data were reviewed by the principal investigators using a designed chart. Thus, all cultured sample of the patient and appropriate information were retrieved from the microbiology laboratory unit registration book. Age, sex, specimen type, bacterial isolates and antibiotic susceptibility pattern were collected using a data extraction sheet. Those with incomplete data that means a data which was not recorded for either of the isolated organism, antibiotics susceptibility, unknown sample type, sex and age were excluded. Statistical package for social sciences (SPSS) version 20 was used for both data entry and analysis. Descriptive statistics were used to determine the frequency of isolated bacteria.

The microbiological lab performs culture and sensitivity test from the urine, puss, blood, ear discharge, eye swab, genital swab, stool, cerebrospinal fluid (CSF), sputum and nasal swab sample suspected for any bacterial infection based on the standard operating procedure (SOP). The samples were sent from different wards however the samples address was not recorded in the laboratory book. All samples were cultured on appropriate culture media i.e. manual blood culture was conducted whenever a blood-stream infection is suspected in trypticase soy broth prepared in the laboratory. If there is an indication of growth like hemolysis, gas, and turbidity the inoculum was subcultured on appropriate solid medium for further identification. Blood and MacConkey agar were used to culture non-fastidious bacteria and Chocolate agar included for fastidious bacteria and Thayer Martin agar for the genital sample was used. The single bacterial colony from culture media was taken for gram staining. Based on the gram reaction biochemical tests selected. Gram-positive bacteria are identified using catalase, coagulase, bacitracin, pyrrolidonyl arylamidase (PYRase), optochin bile solubility and Novobiocin. Gram-negative bacterial was identified based on serial biochemical reactions and fermentation of carbohydrates i.e. oxidase, catalase, triple sugar iron agar, citrate utilization test, urease, lysine iron agar, sulphur indole motility, mannitol fermentation, and indole test. Gram staining and colony characteristic were used for preliminary identification of the bacteria. Some bacterial were identified to species level by biochemical and some of them to genus level by morphological characteristics (grams stain and colony characteristics). The antibiotic sensitivity pattern was determined with the Kirby-Bauer disc diffusion technique and interpreted based on the current clinical and laboratory standard Institute (CLSI) 2014–2017 guideline.

The susceptibility testing was conducted based on the recommendation of the CLSI. Different antibiotic discs (Abtek LTD, UK) were used: Ampicillin (AMP)(10 μg), gentamicin-Gen(10 μg), ciprofloxacin-CRP(5 μg), ceftriaxone-CRT(30 μg), ceftazidime-CAZ(30 μg), norfloxacin-NOR)(10 μg), nitrofurantoin-NIT(300 μg), augmentin-AUG(20/10 μg), cotrimoxazole-COT(1.25/23.75 μg), chloramphenicol-CAF(30 μg), meropenem-MER(10 μg), tetracycline-TAT(30 μg), penicillin G-PEN(10 IU), Clindamycin-CLD(2 μg) and erythromycin-ERY(15 μg).

### Quality control

The microbiology lab performs quality control of all culture system by using *E. coli* (ATCC-25922), *S. aureus* (ATCC- 25923) and *P. aeruginosa* (ATCC-27853) which was ordered and supervised by the national lab of Ethiopia.

### Ethical consideration

Ethical clearance was obtained from, Institutional Review Board (IRB) of Hawassa University College of Medicine and Health Sciences. Besides official permission was obtained from the hospital administration to collect the information from the registration book. Patients’ data were anonymized and kept confidential throughout this study. All data obtained in the course of the study were reserved confidential and used only for this study.

## Results

### Percentage of total isolates within age and sex

A total of 693 bacteria were included for the study from different clinical specimens (blood, urine, puss, genital swab, eye swab, ear discharge, sputum, nasal swab, body fluids, CSF and stool). Of these 435(62.8%) were gram-negative bacteria (GNB) and the rest 258(37.2%) were gram-positive bacteria (GPB). Most of the isolates were detected in children less than 5 years of age (48.2%; 334/693) followed by 24–64 years of age (21.2%; 147/693); the least number of isolates were found from patients > 64 years of age. Most of the isolates were from male patients with a male to female ratio 1.13:1 (Table 1).

**Table 1:** Age and sex distribution of patients with bacterial isolates, HUCSH, Southern Ethiopia

Age group in year	Sex		Total Bacteria (%)
	Male N (%)	Female N (%)	
< 5	189(51.2)	145(44.8)	334(48.2)
5–14	62(16.8)	68(21)	130(18.8)
14–24	33(8.9)	29(9)	62(8.9)
24–64	74(20.1)	73(22.5)	147(21.2)
> 64	11(3)	9(2.8)	20(2.9)
Total	369(100)	324(100)	693(100)

### Sample type and bacterial isolates

Regarding sample type, most of the isolates were from urine sample 251(36.2%) followed by puss sample 190(27.4%) and blood 131(18.9%). When we observe the frequency of isolates; *S. aureus* was the leading 156(22.5%) and *S. pneumoniae* 7(1%) was the least from gram-positive bacteria. *Klebsiella spp* 154(22.2%) were the predominant isolates and *N. gonorrhoea* 3(0.4%) was the least from gram-negative bacteria (Table 2).

**Table 2:** Distribution of sample type and bacterial isolates from a study of the burden of antimicrobial resistance at HUCSH, southern Ethiopia

				Specimen type								Total
Isolates	Urine	Body Fluids	Stool	Ear Swab	Eye swab	CSF	Blood	Sputum	Puss	Nasal swab	Genital swab	
*S. auraes*	22(8.8)	6(31.6)	*0*	5(11.9)	6(50)	0	19(14.5)	1(5.9)	97(51.1)	0	0	156(22.5)
*CoNS*	9(3.6)	0	0	0	0	0	55(42)	0	0	0	0	64(9.2)
*Enterococcus spp*	17(6.8)	1(5.3)	*0*	0	1(8.3)	0	10(7.6)	1(5.9)	1(0.5)	0	0	31(4.5)
*S. pneumonia*	0	0	0	3(7.1)	0	1(14.3)	3(2.3)	0	0	0	0	7(1)
*Klebsiella spp*	79(31.5)	5 (26.3)	0	9(21.4)	2(16.7)	0	26(19.8)	8(47.1)	25(13.2)	0	0	154(22.2)
*E. coli*	70(27.9)	2(10.5)	0	9(21.4)	1(8.3)	2(28.6)	5(3.8)	0	26(13.7)	1 (50)	0	116(16.7)
*Pseudomonas spp*	11(4.4)	1(5.3)	0	9(21.4)	0	0	7(5.3)	3(17.6)	15(7.9)	1 (50)	0	47(6.8)
*Citrobacter spp*	16(6.4)	3(15.8)	0	2(4.8)	1(8.3)	0	1(0.8)	2(11.8)	7(3.7)	0	0	32(4.6)
*Enterobacter spp*	16(6.4)	0	0	0	1(8.3)	0	2(1.5)	1(5.9)	4(2.1)	0	0	24(3.5)
*Acinetobacter spp*	4(1.6)	0	0	1(2.4)	0	0	2(1.5)	1(5.9)	10(5.3)	0	0	18(2.6)
*Proteus Spp*	6(2.4)	1(5.3)	0	4(9.5)	0	0	0	0	5(2.6)	0	0	16(2.3)
*Shigella spp*	0	0	12(63.2)	0	0	0	0	0	0	0	0	12(1.7)
*Salmonella spp*	1(0.4)	0	7(36.8)	0	0	0	1(0.8)	0	0	0	0	9(1.3)
*N. meningitis*	0	0	0	0	0	4(57.1)	0	0	0	0	0	4(0.6)
*N. gonorrhea*	0	0	0	0	0	0	0	0	0	0	3(100)	3(0.4)
*Total*	251(36.2)	19(2.7)	19(2.7)	42(6.1)	12(1.7)	7(1)	131(18.9)	17(2.5)	190(27.4)	2(0.3)	3(0.4)	693(100)

### Antibiotics resistance patterns of GPB

Concerning antibiotics resistance, we can say that extraordinarily resistant were recorded for cotrimoxazole, penicillin G, norfloxacin and ceftazidime by most of the gram-positive bacteria. In the other hand least resistance was recorded for nitrofurantoin (Table 3).

**Table 3:** Antibiotics resistance patterns of GPB, HUCSH, and Southern Ethiopia

Antibiotics	*S. aureus* (156)	*CoNS (64)*	*Enterococcus spp(31)*	*S. pneumoniae (7)*	Total
	R (%)	R (%)	R (%)	R (%)	R (%)
AMP	NR	NR	20(74.1)	NR	20(74.1)
GEN	42(31.8)	32(55.2)	NR	NR	74(38.9)
COT	61(73.5)	49(90.7)	NR	4(66.7)	116(81.1)
PEN	41(80.4)	24(92.3)	6(75)	1(33.3)	72(81.8)
CAF	22(19.5)	22(40.7)	8(34.8)	3(60)	55(28.2)
CLD	2(5.3)	ND	NR	NR	2(5.3)
NOR	15(65.2)	3(33.3)	10(83.3)	NR	28(63.6)
NIT	1(14.3)	ND	1(12.5)	NR	2(13.3)
ERY	20(31.7)	9(50)	11(73.3)	0(0)	40(40.4)
TAT	11(61.1)	ND	3(100)	0(0)	14(63.6)
CIP	42(31.8)	27(46.6)	19(76)	NR	88(40.9)
AUG	51(52)	24(39.3)	NR	NR	75(47.2)
CTR	39(45.3)	22(41.5)	NR	NR	61(43.9)
CAZ	27(58.7)	31(79.5)	NR	ND	58(68.2)

*NR*- Not recommended, *ND*-not done, *R*-Resistance, *T*- total tested, *CoNS*- *coagulase-negative staphylococcus,* ampicillin (AMP)(10 μg), gentamicin-Gen(10 μg), ciprofloxacin-CRP(5 μg), ceftriaxone-CRT(30 μg), ceftazidime-CAZ(30 μg), norfloxacin-NOR)(10 μg), nitrofurantoin-NIT(300 μg), augmentin-AUG(20/10 μg), cotrimoxazole-COT(1.25/23.75 μg), chloramphenicol-CAF(30 μg), meropenem-MER(10 μg), tetracycline-TAT(30 μg), penicillin G-PEN(10 IU), Clindamycin-CLD(2 μg) and erythromycin-ERY(15 μg).

### Antibiotics resistance patterns of Enterobacteriaceae

Again higher resistance was reported from Enterobacteriaceae. As shown in Table 4 ampicillin (92.5%), cotrimoxazole (85%) and tetracycline (85%) was substantially resisted antibiotics.

**Table 4:** Antibiotics resistance patterns of Enterobacteriaceae, HUCSH Southern Ethiopia

Antibiotics	*E. coli (*116*)*	*Klebsiella spp (154)*	*Enterobacter spp (24)*	*Citrobacter spp* (32)	*Proteus spp* (16)	*Shigella spp* (12)	*Salmonella spp* (9)	Total
	R (%)	R (%)	R (%)	R (%)	R (%)	R (%)	R (%)	R (%)
AMP	84(92.3)	106(93.8)	17(94.4)	21(91.3)	8(88.9)	6(75)	5(100)	247(92.5)
GEN	55(55.6)	107(79.3)	15(78.9)	16(61.5)	7(50)	4(40)	3(50)	207(67)
CRP	51(48.1)	57(41.3)	8(38.1)	6(22.2)	5(55.6)	2(25)	3(33.3)	132(41.5)
CTR	50(66.7)	97(86.6)	10(83.3)	14(63.6)	8(61.5)	5(50)	4(50)	188(74.6)
CAZ	28(56)	46(78)	4(66.7)	3(37.5)	2(40)	2(100)	1(100)	86(65.6)
NOR	46(61.3)	35(43.8)	8(47.1)	6(31.6)	4(66.7)	ND	–	100(50.8)
NIT	1(3.8)	1(7.1)	1(11.1)	1(16.7)	1(100)	ND	0(0)	5(8.7)
AUG	65(65.7)	93(74.4)	14(82.4)	15(60)	5(38.5)	4(50)	4(57.1)	200(68)
COT	51(81)	86(91.5)	14(82.4)	6(54.5)	5(83.3)	4(80)	4(100)	170(85)
CAF	26(36.6)	51(51)	5(50)	8(42.1)	4(50)	3(42.9)	3(42.9)	100(45.1)
MER	0(0)	3(13.6)	5(62.5)	2(42.9)	0(0)	ND	0(0)	11(22.9)
TAT	6(100)	4(100)	3(60)	4(100)	0(0)	ND	ND	17(85)

*ND*-Not done, *R*-Resistance, ampicillin (AMP)(10 μg), gentamicin-Gen(10 μg), ciprofloxacin-CRP(5 μg), ceftriaxone-CRT(30 μg), ceftazidime-CAZ(30 μg), norfloxacin-NOR)(10 μg), nitrofurantoin-NIT(300 μg), augmentin-AUG(20/10 μg), cotrimoxazole-COT(1.25/23.75 μg), chloramphenicol-CAF(30 μg), meropenem-MER(10 μg), tetracycline-TAT(30 μg)

### Antibiotics resistance patterns of gram negatives non-Enterobacteriaceae

Likewise, a remarkable resistance for tested antibiotics was recorded by most non- Enterobacteriaceae isolates. As shown in Table 5 tetracycline (100%), cotrimoxazole (93.1%) and ampicillin (92.9%), was mainly resisted antibiotics.

**Table 5:** Antibiotics resistance patterns of non- Enterobacteriaceae group at HUCSH, Southern Ethiopia

Antibiotics	*Pseudomonas spp* (47)	*Acinetobacter spp* (18)	*N. gonorrhoea* (3)	*N. meningitis* (4)	Total
	R (%)	R (%)	R (%)	R (%)	R (%)
Amp	23(92)	15(88.2)	NR	ND	39(92.9)
Gen	22(55.2)	11(84.6)	NR	ND	33(62.2)
CRP	14(32.6)	9(56.2)	2(100)	2(50)	27(41.5)
CTR	23(67.6)	10(76.9)	2(66.7)	1(100)	38(71.7)
CAZ	10(47.6)	3(75)	1(100)	1(100)	15(55.6)
NOR	3(25)	3(60)	NR	ND	7(41.2)
AUG	24(85.7)	11(78.6)	NR	1(100)	36(83.7)
COT	15(88.2)	10(100)	NR	1(50)	27(93.1)
CAF	14(70)	10(83.3)	NR	2(100)	26(76.5)
MER	2(50)	2(50)	NR	ND	4(50)
TAT	3(100)	2(100)	ND	NR	5(100)

ND-not done, NR-not recommended, ampicillin (AMP)(10 μg), gentamicin-Gen(10 μg), ciprofloxacin-CRP(5 μg), ceftriaxone-CRT(30 μg), ceftazidime-CAZ(30 μg), norfloxacin-NOR)(10 μg), nitrofurantoin-NIT(300 μg), augmentin-AUG(20/10 μg), cotrimoxazole-COT(1.25/23.75 μg), chloramphenicol-CAF(30 μg), meropenem-MER(10 μg), tetracycline-TAT(30 μg).

### Multi-drug resistance pattern

**Fig. 1:**
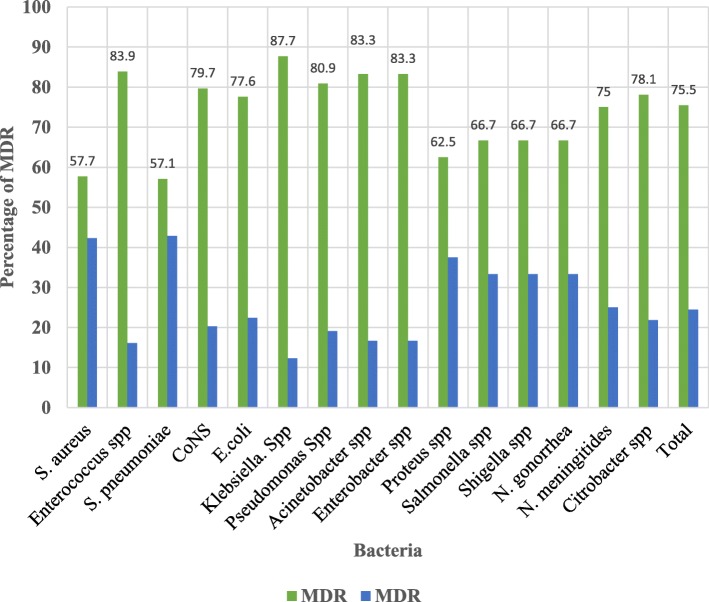
Multi-drug resistance in a study of the burden of antimicrobial resistance at HUCSH

Multi-drug resistance (MDR) is defined as resistance to at least one or more agents in three or more antibiotics categories [12]. Overall MDR to all isolates were (75.5%). Of this *Klebsiella spp* had been with highest MDR which was (87.7%) followed by *Enterococcus spp* (83.9%), *Acinetobacter spp* (83.3%), *Enterobacter spp* (83.3%) and *Pseudomonas spp* (80.9%). Least MDR was identified from *S. aureus* (57.7%) followed by *S. pneumoniae* (57.1%), *Proteus spp* (62.5%) and (66.7%) for each of these isolates (*Salmonella spp*, *Shigella spp* and *N. gonorrhoea*) (Fig.1).

## Discussion

Antimicrobial resistance is one of the most serious problems faced the world nowadays. The lack of effective antibiotics will challenge healthcare workers to combat infectious diseases including their potential to manage complications, especially among immunocompromised clients. In this study, gram-negative bacteria were predominantly isolated from most specimens. The finding is similar to the studies reported from Southwest and Debre Markos. Ethiopia reported regardless of specimen type difference [13, 14]. The most frequently isolated gram-positive bacterium was *S. aureus* and the lest frequently isolated bacterium was *S. pneumoniae which* is in-line with a study conducted in Debre Markos on the clinical specimen and Bahir Dar from nosocomial infected patients in Ethiopia [14, 15] and in India from clinical specimen [16]. In the other hand *Klebsiella, spp* were the predominant gram-negative bacteria in our study, in-line with our finding studies from India reported the same [17]. In contrast to our study, *E. coli* were reported as dominant bacteria from India from clinical specimen conducted retrospectively [16] which can be explained by study area, period and individual difference.

Our finding indicated that extraordinary rates of resistance for most antibiotics. Specifically, gram-positive bacteria were resistant to ampicillin, cotrimoxazole and penicillin G which is in agreement with reports from Debre Markos [14] and Addis Ababa, Ethiopia [18], Ardabil, Iran [19], Gabon, Central Africa [10] and South East Nigeria [20]. Even though there is a difference in the study area and period, the amount of sample analyzed and a number of bacterial isolates captured.

Gram-negative bacteria had a remarkable rate of resistance to most antibiotics other studies from Addis Ababa, Ethiopia [21], Saudi Arabia [22], and Libya [23] agreed with our study as gram-negative bacteria (GNB) are more resisted as compared to GPB. On the other hand, low rates of resistance for all Enterobacteriaceae was observed to nitrofurantoin and meropenem in-line with studies from Bahir Dar, Ethiopia [24], Benin Nigeria [25].

Concerning non- Enterobacteriaceae, a high rate of resistance were observed to tetracycline, cotrimoxazole, ampicillin, amoxicillin-clavulanic acid, chloramphenicol and gentamicin. This resistance may be due to *Pseudomonas spp* and *Acinetobacter spp* which are the most common known resistant because of their nature of resistance for different chemical agents. Other studies supported this finding as high resistance Pseudomonas Spp from Iran [26], Dhaka [27], Nigeria [28], and Taiwan [29].

In our study we identified (57.1–87.3%) MDR, this showed that there are a few options to treat patients in the study area. Which was almost similar to others finding like, Studies reported from Mexico [30], Ethiopia [31], Hungary [32]. In general, we can say that antibiotics resistance will be a great challenge in the study area if there is no appropriate solution set on time.

### Limitation of the study

 ✓ This study is a retrospective study so could not explain the current antimicrobial patterns in the study area.

✓ The study did not determine the resistance detected was due to hospital-acquired or community acquired infection.

✓ The study did not show the trends of antibiotics resistance from year to year.

## Conclusions

This retrospective study identified revealed that nearly all the isolated bacteria developed substantial rates of resistance to most of the antibiotics that are frequently used in the study area. To some extent, gentamicin, ciprofloxacin, clindamycin, nitrofurantoin and chloramphenicol were effective to treat GPB, whereas penicillin G, cotrimoxazole, augmentin, ampicillin, norfloxacin, ceftazidime and tetracycline were the least effective antibiotics. Therefore, in general, the antibiotic-resistant rate is high in the specified study area, the physician should prescribe antibiotics based on drug susceptibility report. In case of the empirical treatment is mandatory prescribing antibiotics with less resistance may be helpful to manage bacterial infections. We hope also that these studies finding will help policymakers nationally as the best data for developing intervention measures and to exclude most resistant drugs from the market.

## Data Availability

The datasets used and/or analysed during the current study are available from the corresponding author on reasonable request.

## Abbreviations

AMR: Antimicrobial Resistance
ATCC: American type culture collection
CLSI: Clinical and laboratory standard Institute
CoNS: *Coagulase-negative staphylococcus*
CSF: Cerebrospinal fluid
ESBL: Extended-spectrum ß-lactamase
GNB: Gram-negative bacteria
GPB: Gram-positive bacteria
HUCSH: Hawassa University Comprehensive Specialized Hospital
IRB: Institutional Review Board
MDR: Multi-drug resistance
MRSA: Methicillin-resistant *S. auraes*
PYRase: Pyrrolidonyl arylamidase
SNNPR: Southern Nation Nationalities’ and peoples’ region
SOP: Standard operating procedure
SPSS: Statistical package for social sciences
